# Animal multicellularity and polarity without Wnt signaling

**DOI:** 10.1038/s41598-017-15557-5

**Published:** 2017-11-13

**Authors:** Quentin Schenkelaars, Marine Pratlong, Laurent Kodjabachian, Laura Fierro-Constain, Jean Vacelet, André Le Bivic, Emmanuelle Renard, Carole Borchiellini

**Affiliations:** 1 Aix Marseille Université, CNRS, IRD, IMBE UMR 7263, Avignon Université, Institut Méditerranéen de Biodiversité et d’Ecologie marine et continentale, Station Marine d’Endoume, Marseille, France; 2 Department of Genetics and Evolution, University of Geneva, Sciences III, 30 Quai Ernest Ansermet, CH-1211 Geneva 4, Switzerland; 3 Aix Marseille Université, CNRS, Centrale Marseille, I2M, Equipe Evolution Biologique et Modélisation, Marseille, France; 4 Aix Marseille Université, CNRS, Institute of Developmental Biology of Marseille (IBDM), case 907, 13288 Marseille cedex 09, France

## Abstract

Acquisition of multicellularity is a central event in the evolution of Eukaryota. Strikingly, animal multicellularity coincides with the emergence of three intercellular communication pathways – Notch, TGF-β and Wnt – all considered as hallmarks of metazoan development. By investigating *Oopsacas minuta* and *Aphrocallistes vastus*, we show here that the emergence of a syncytium and plugged junctions in glass sponges coincides with the loss of essential components of the Wnt signaling (i.e. Wntless, Wnt ligands and Disheveled), whereas core components of the TGF-β and Notch modules appear unaffected. This suggests that Wnt signaling is not essential for cell differentiation, polarity and morphogenesis in glass sponges. Beyond providing a comparative study of key developmental toolkits, we define here the first case of a metazoan phylum that maintained a level of complexity similar to its relatives despite molecular degeneration of Wnt pathways.

## Introduction

**Figure 1:**
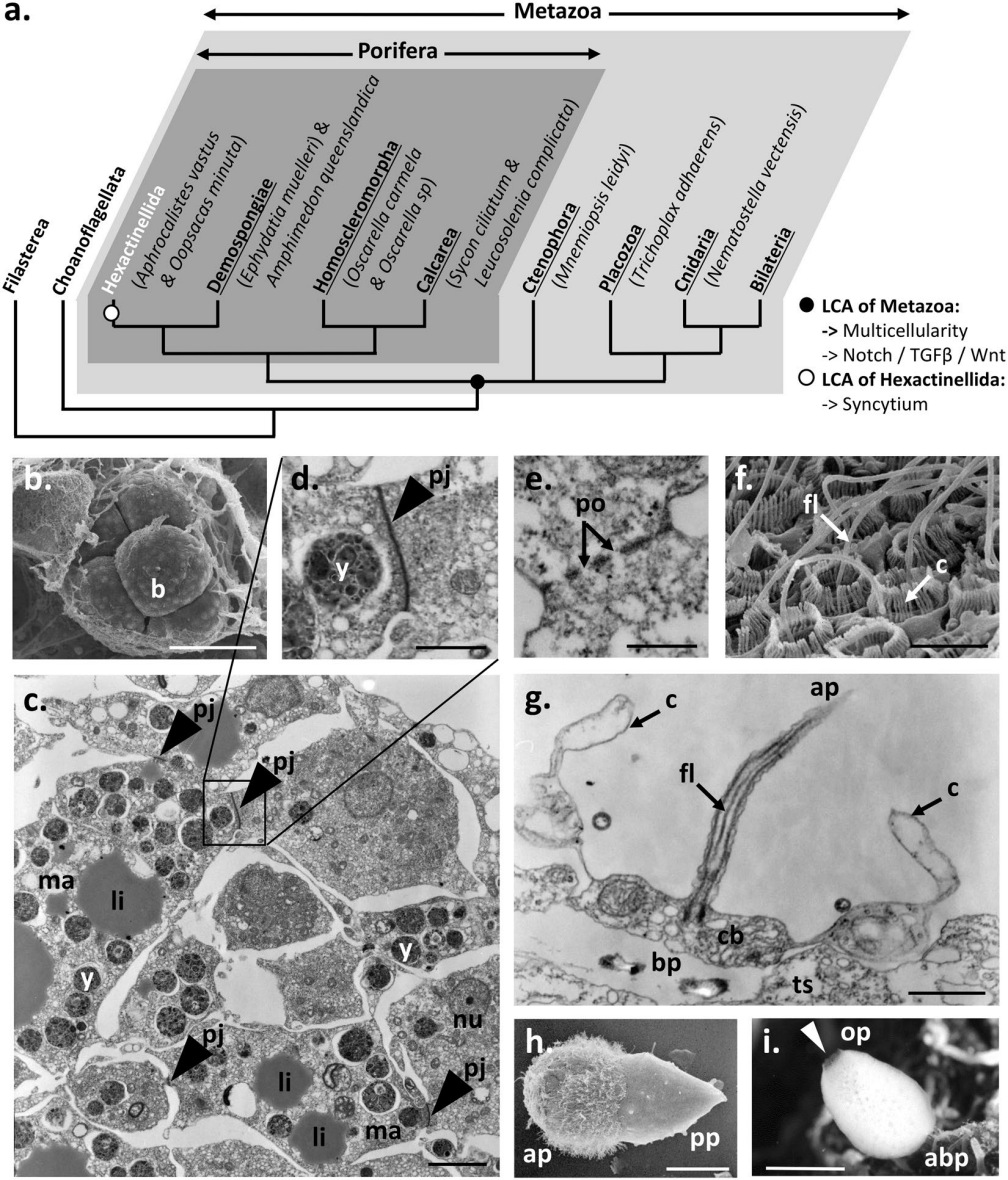
Peculiar cell organization and development of glass sponges: *Oopsacas minuta*. (**a**) Phylogenetic position of glass sponges (Hexactinellida) among poriferans and metazoans. LCA: Last common ancestor. (**b**.,**f**. and **h**.) Scanning electron micrographs. (**c**.,**d**.,**e**. and **g**.) Transmission electron micrographs of epoxy sections. (**b**.) 8-cell stage embryo within maternal tissue^43^. (**c**. and **d**.) Details of embryo showing the fusion of macromeres and the establishment of plugged junctions. (**e**.) Cytoplasmic continuity between cells through plugged junction. (**f**. and **g**.) Collar bodies evidencing a clear cell polarity as shown by the apical pole, ap and the basal pole, bp. (**h**) Larva showing a clear anterior-posterior axis. The anterior pole and posterior pole are indicated: ap and pp respectively. (**i**) Adult specimen harboring a clear apico-basal axis. The oral pole (apical pole) and aboral pole (basal pole) are indicated op and abp, respectively. Abbreviations: b, blastomere; c, collar; cb, collar bodies; fl, flagellum; li, lipid inclusion; ma, macromere; nu, nucleus; os, osculum (white arrowhead); pj, plugged junction (grey arrowhead); po, pore particles; ts, trabecular syncytium; y, yolk. Scale: (**b**.), 43 µm; (**c**.), 3 µm; (**d**.,**e**. and **g**.), 1 µm; (**f**.), 4.3 µm; (**h**.), 50 µm; (**i**.), 0.5 cm.

**Figure 2:**
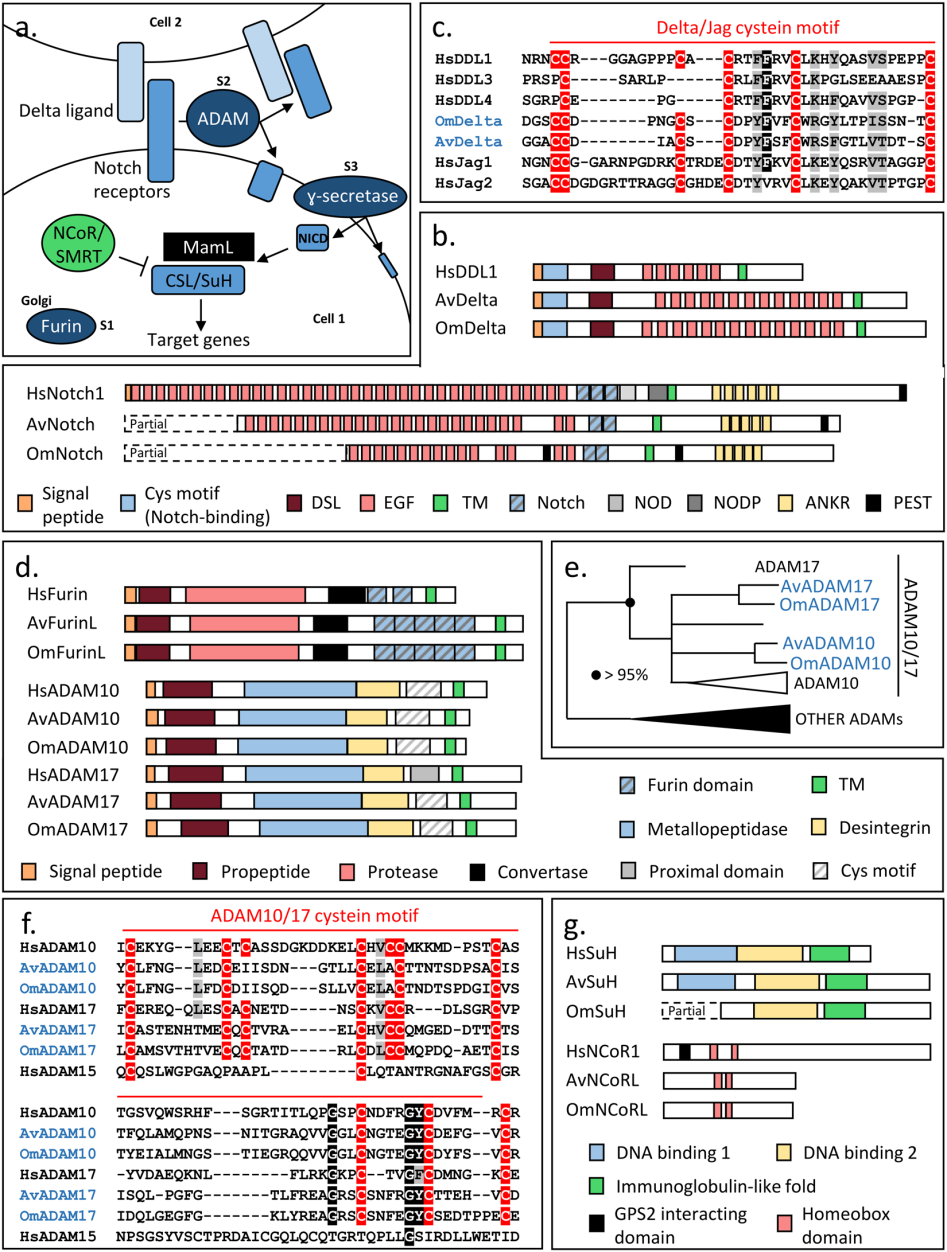
Conservation of the Notch pathway in glass sponges. (**a**.) Schematic representation of the Notch pathway. In blue the components retrieved in glass sponges, in green the component retrieved in glass sponges but not described in other poriferan lineages until now and in black the element absent in glass sponges and other poriferan. The three successive cleavages of Notch are indicated: S1, S2 and S3. (**b**.) Domain composition of Notch and Delta proteins. (**c**.) Alignment of the Delta/Jag cysteine motif. (**d**.) Domain composition of Furin and ADAM proteins. (**e**.) Phylogenetic analysis revealing two ADAM10/17 in glass sponges. Black and white triangles represent clades of proteins with a node support higher than 95% and lower than 70%, respectively. (**f**.) Conservation of the proximal domain of ADAM as shown by the conservation of the cysteine motif. (**g**.) Domain composition of SuH and CoR1.

**Figure 3:**
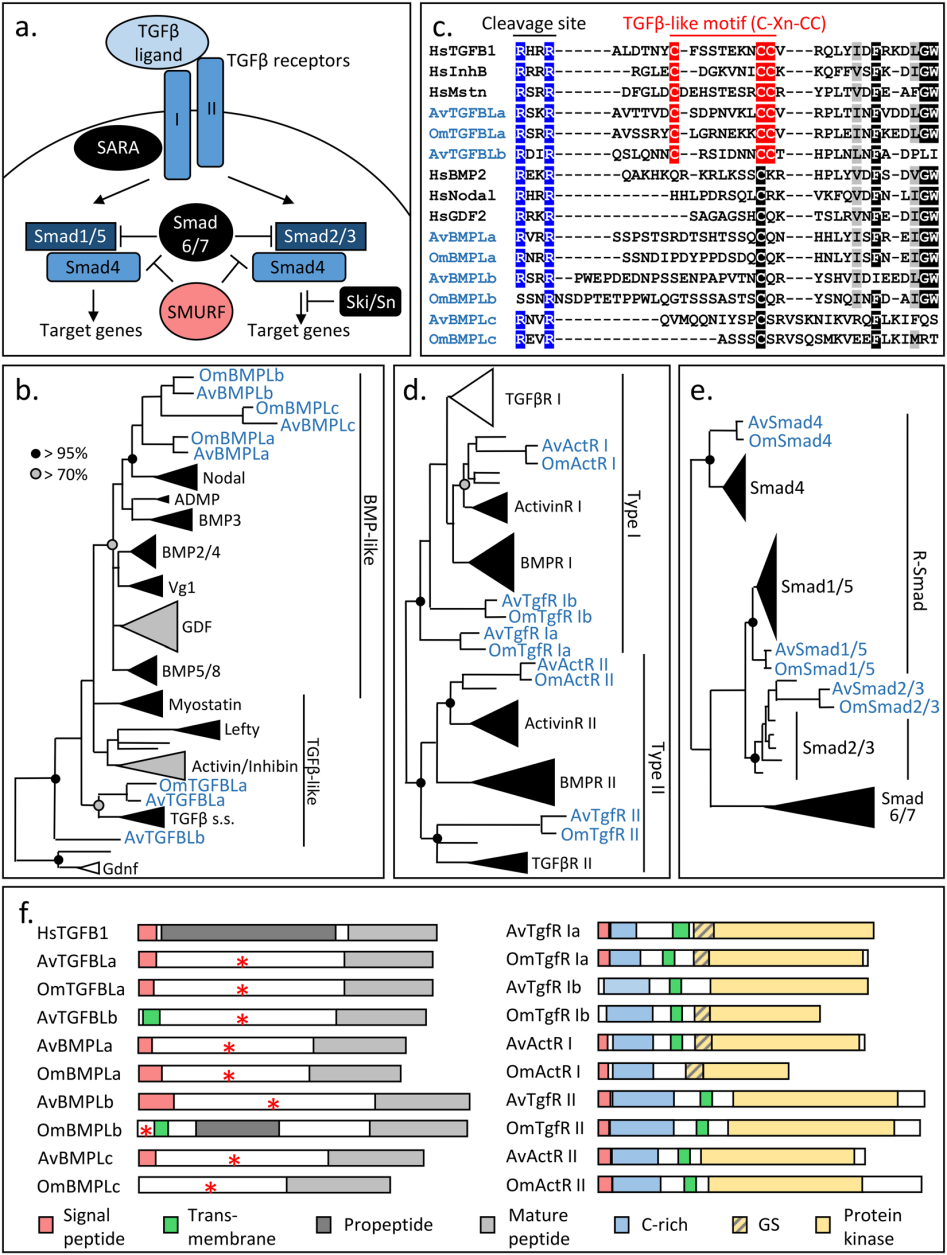
Conservation of the TGF-β pathway in glass sponges. (**a**.) Schematic representation of the TGF-β pathway. In blue the components retrieved in glass sponges, in black the element absent in glass sponges and other poriferan and in red the component retrieved in other sponges but not in glass sponges. (**b**.) Phylogenetic analysis revealing three BMP-like, one TGFβ-like and one unclassified TGF ligand in glass sponges. (**c**.) Alignment of TGFβ-like motif. In blue the RXXR cleavage site. (**d**.) Phylogenetic analysis revealing three type I and two type II TGFβ receptors in glass sponges. (**e**.) Phylogenetic analysis revealing Smad1/5, Smad2/3 and Smad4 in glass sponges. (**b**.,**d**. and **e**.) Black, grey and white triangles represent clades of proteins with a node support higher than 95%, comprise between 70% and 95% or lower than 70%, respectively. (**f**.) Domain composition of TGFβ ligands and receptors.

**Figure 4:**
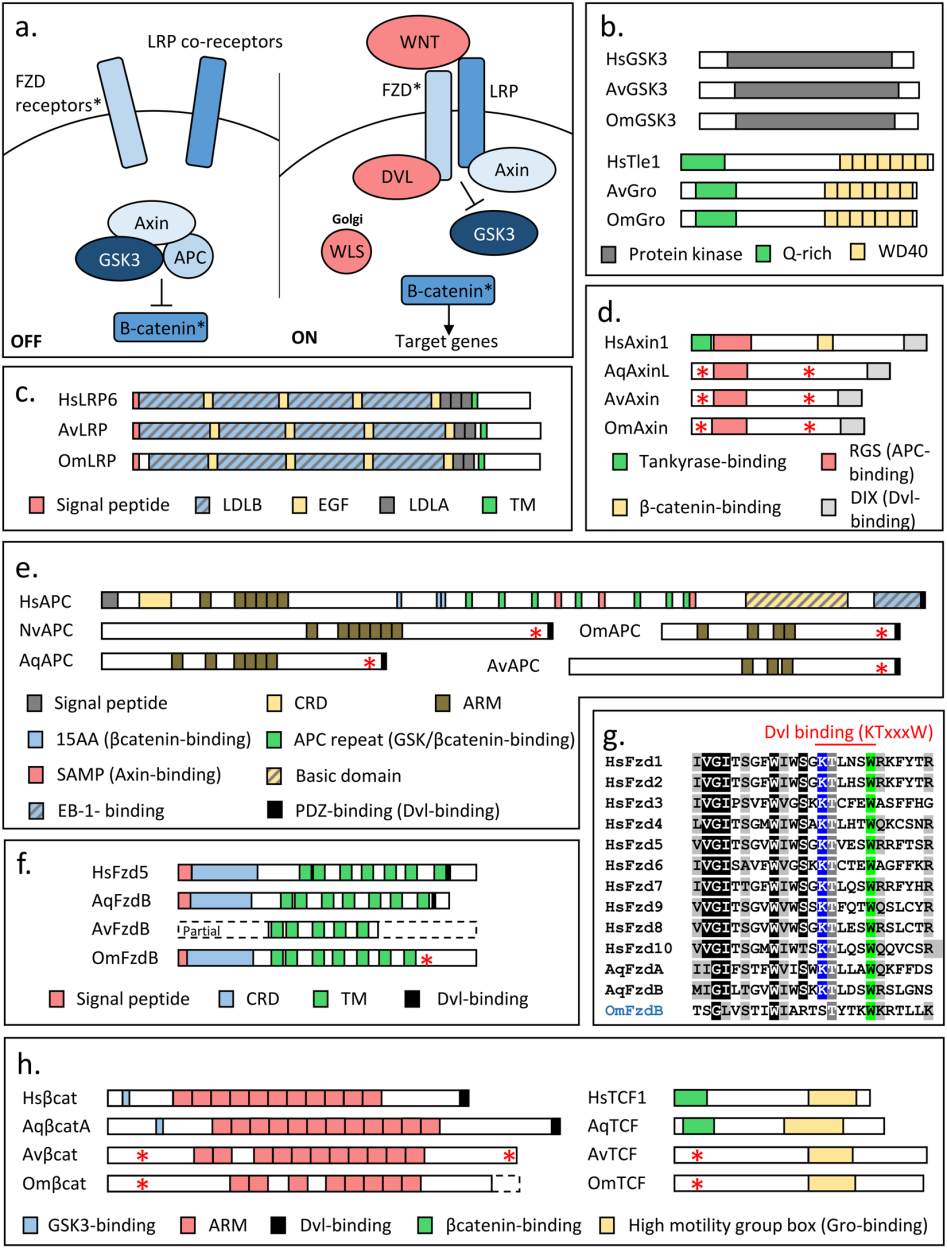
Loss of the Wnt pathway in glass sponges. (**a**.) Schematic representation of the Wnt pathway. In blue the components retrieved in glass sponges and in red the components retrieved in other sponges but not in glass sponges. Black asterisks mean that the proteins were retrieved but that they show unusual domain compositions. (**b**.) Domain composition of GSK3 and Groucho. (**c**.) Domain composition of LRP. (**d**.) Domain composition of Axin. Note the two missing domains in poriferan sequences (**e**.) Domain composition of APC. Note that the protein in porifera and cnidarian are truncated (**f**.) Domain composition of Frizzled. Note the absence of the Dvl-bonding motif (**g**.) Alignment of the Dvl-bonding motif. Note the absence of the first residue in glass sponges. (**h**.) Domain composition of β-catenin and TCF. Note the absence of the GSK3-binding domain and Dvl-binding motif in glass sponge β-catenin and the absence of Groucho-binding domain in TCF. (**d**.,**e**.,**f**. and **h**.) Red asterisks correspond to missing domains when proteins are compared to the human sequence. Abbreviation: Aq, *Amphimedon queenslandica* and Nv, *Nematostella vectensis*.

The emergence of multicellularity was a critical step in the origin of life diversity^1^. Multicellular lifestyles appeared independently at least 25 times from diverse single-celled ancestors^1^. However, few lineages accomplished more complex organizations with specialized tissues and/or organs: fungi, land plants, red algae, brown algae and animals^2–4^. It is commonly accepted that the animal (metazoan) multicellular ancestor likely resulted from the failure of daughter cells to separate after division (colonial hypothesis)^2^. However, little is known about the mechanisms that allowed the stable transition towards multicellularity. Until now, hints were gleaned through comparison of the genomes of living animals with those of their closest unicellular relatives **(**Fig. 1a **)**, namely Choanoflagellata and Filasterea^5–8^. In contrast to animals in which multicellularity is obligate, only few species of choanoflagellates and Filasterea are able to transiently form colonies and aggregative stages^1,7,9–12^. Thus, comparisons of the genetic toolkits of these predominantly unicellular lineages to those of animals, have identified numerous genetic innovations that are now considered as hallmarks of animal multicellularity. Among them, the Delta/Notch pathway **(**Fig. 2a **)** is mainly considered as a key player in cell fate decision while the TGF-β/TGFR **(**Fig. 3a **)** and the Wnt/Frizzled **(**Fig. 4a **)** modules mediate patterning by controlling cell proliferation and cell motility^6,13–18^. Co-occurrence of these three ligand/receptor interactions suggests that empowering cell cooperation and cell specialization by efficient cell-to-cell communication was certainly a decisive step in the acquisition of stable multicellularity in animals.

Among animals (metazoans), glass sponges (Porifera, Hexactinellida) have a very peculiar tissue organization^19^, which makes them highly relevant to address the evolution of cell-to-cell communication during morphogenesis. Like most metazoans, glass sponges begin life through successive cell divisions **(**Fig. 1b **)**, which give rise to a hollow blastula made of 16 to 32 cells. However, after the fifth division, cleavages become unequal, cytoplasmic bridges (named plugged junctions or plugs) are formed between blastomeres, and macromeres progressively merge together giving rise to a syncytial larva **(**Fig. 1c and d **)** ^20^. Adult glass sponges mainly consist of a multinucleated syncytium as well (the trabecular syncytium), which is connected to different cell types (i.e. archeocytes, choanoblasts and collar bodies) via plugged junctions **(**Fig. 1e–g **)** ^19^. This unique syncytial feature, acquired more than 545 million years ago in the last common ancestor of glass sponges^21^, raises exciting questions regarding its consequences on the developmental toolkit of extant species.

In order to test the universality of the classical cell-to-cell communication modules of animal development (i.e. Notch, TGF-β and Wnt pathways), we analyzed the transcriptomes of two distantly related glass sponges *Oopsacas minuta* Topsent, 1927 (Hexactinellida, Lyssacinosida) and *Aphrocallistes vastus* Schulze, 1886 (Hexactinellida, Hexactinosida) and compared them to those of other sponge lineages (i.e. Calcarea, Demospongiae and Homoscleromorpha) and of other metazoans **(**Fig. 1a **)**.

## Results

### Conserved Notch pathway

In contrast to the previous analysis of *A. vastus* transcriptome^22^, we identified the Notch ligand Delta in glass sponges. Indeed, consistent with the analysis of *Oscarella carmela*, a unique Delta protein was retrieved in glass sponge, whereas five were previously described in *Amphimedon* genome^23^. This suggests that a single ligand was certainly present in the last common ancestor of sponges and that other paralogs arose later by lineage specific duplications. Computational domain analyses allowed us to confirm the conservation of the overall structure of glass sponge Delta proteins **(**Fig. 2b **)**. Indeed, the DSL domain, several EGF domains and the transmembrane region were predicted by Interproscan. Intriguingly, the Notch-binding domain of Delta/Jagged proteins (i.e. MNLL) is not predicted in sponges except in one of the five proteins retrieved in *Amphimedon* ^23^. However, this protein does not show a typical composition, as it lacks EGF motifs. In this context, we draw attention on a pattern of cysteines conserved between MNLL and the N-terminus of all poriferan sequences **(**Fig. 2c **)**. Since cysteines are extremely conserved only when involved in functional interactions^24^, it likely reflects the conservation of the MNLL. As we found *bona fide* Notch proteins as well (i.e. several EGF, Notch domains, one transmembrane domain, five ANKR and at least one PEST domain) **(**Fig. 2b **)**, it appears that Delta/Jagged activation and signal transduction through the membrane may occur in glass sponges. Functional studies are however required to prove ligand/receptor interactions in poriferans and to identify key residues.

We also identified in glass sponges all the proteins involved in the multiple cleavages of the Notch receptor. We retrieved Furin **(**Fig. 2d **)**, two ADAM10/17 proteins **(**Fig. 2d and e **)** and the four ɣ-secretase subunits (i.e. Presenilin, Presenilin Enhancer 2, Anterior Pharynx defective I and Nicastrin) **(**Supplementary Table 1 **)**. Interestingly, these proteins, required for S1, S2 and S3 cleavages respectively, show a similar domain composition to their human orthologs **(**Fig. 2d and f **)**. Thus, we assume that Notch processing and the release of the NICD signaling fragment is possible in glass sponges.

Finally, glass sponges also possess the CSL/SuH transcription factor and a protein sharing similarities with the NCoR/SMRT co-repressor **(**Fig. 2g **)**, whereas in agreement with previous analyses of the pathway in Demospongiae and Homoscleromorpha^23^, no MAML protein was retrieved. Thus, all the core proteins required for Notch pathway activity are present in glass sponges **(**Fig. 2a **)**.

### Conserved TGFβ pathway

By dissecting the diversity of the TGFβ core components in early branching metazoans (i.e. *Nematostella*, *Mnemiopsis*, *Trichoplax* and *Amphimedon*) **(**Table 1 **)**, previous studies revealed that the number of TGFβ receptors and Smad proteins is relatively stable in these lineages, whereas the number of ligands is highly variable^25^. The genome of *Amphimedon* however challenges this statement by revealing many Smad paralogs. In addition, many TGFβ ligands retrieved in *Amphimedon* and *Mnemiopsis* are still unclassified. Thus, the number of ligands and Smad proteins in the last common ancestor of Metazoa and their evolution throughout animal kingdom remains difficult to assess as poriferans and ctenophores represent the earliest branching metazoan phyla^15,26–28^.

**Table 1:** Number of TGFβ ligands, TGFβ receptors and Smad proteins in non-bilaterian animals.

	Aphrocallistes	Oopsacas	Amphimedon	Mnemiopsis	Trichoplax	Nematostella
**TGFβ ligands**	**5**	**4**	9	9	5	6
BMP-like	**3**	**3**	0 (+7)	2 (+5)	4	4
TGFβ-like	**1 (**+**1)**	**1**	2	2	1	2
**TGFβ receptors**	**5**	**5**	5	4	4	5
Type I	**3**	**3**	3	3	3	3
Type II	**2**	**2**	2	1	1	2
**Smads - Total**	**3**	**3**	10	5	4	4
Smad4	**1**	**1**	3	1	1	1
Smad1/5	**1**	**1**	3	2	1	1
Smad2/3	**1**	**1**	2	1	1	1
Smad6/7	**0**	**0**	0	1	1	1
Unclassified	**0**	**0**	2	0	0	0

Numbers in bold correspond to the data provided by our study. Numbers in parentheses correspond to the proteins classified thanks to the cysteine patterns (single cysteine in BMP-like versus C-Xn-CC motif in TGFβ-like).

We found five TGFβ ligands in glass sponges **(**Fig. 3b and Supplementary Table 2 **)**. One appears to belong to the TGFβ-like sub-family (i.e. TGFβLa) while three others are clustered within BMP-like proteins (i.e. BMPLa, BMPLb and BMPLc). The last one branches basally, making it difficult to assign it to any TGFβ subgroup. Interestingly, the close examination of each sub-family revealed that TGFβ-like proteins show a C-Xn-CC motif (where n is comprised between 6 and 8) between the cleavage site and the mature peptide, whereas BMP-like proteins show a unique cysteine confirming the assignment of glass sponge BMPs to this sub-family **(**Fig. 3c **)**. In contrast, the TGFβ-like motif was found in TGFβLa and in the fifth member, which we propose to name TGFβLb.

Since the TGFβ-like cysteine motif described here appears as an efficient tool to shed light on the evolution of TGFβ ligands when phylogenetic analyses are not informative, we tried to confirm the assignment of *Amphimedon* and *Mnemiopsis* proteins and to decipher whether the unclassified proteins^25^ belong to the BMP-like or the TGFβ-like subfamilies. Through this approach, we suggest that all previously unclassified proteins are BMP-like proteins **(**Table 1 and Supplementary Figure 1). Our analysis reveals that sponges and ctenophores possess several BMP-like proteins and only two TGFβ-like proteins, likely reflecting the situation in the last common ancestor of animals. This finding calls for functional studies to examine the role of these cysteines, which were subjected to a very high pressure of selection across animal evolution.

In addition to TGFβ ligands, three type I receptors (TgfR Ia, TgfR Ib, ActR I), two type II receptors (TgfR II and ActR II) and three Smad proteins (Smad2/3, Smad4 and Smad1/5) were identified **(**Fig. 3d and e **)**, whereas like in the *Amphimedon* genome^25^ we failed to find Smad6/7, SARA, the co-repressor Ski/Sno, but also SMURF in either transcriptomes. Thus, although the number of Smad1/5 paralogs and the presence of Smad6/7 in the last common ancestor of animals remain difficult to assess, the analysis of glass sponge transcriptomes allows us to propose a unique copy of Smad4 and Smad2/3 at the dawn of animals, while several duplications may have occurred in Demospongiae.

At the protein level, domain analyses revealed that despite the absence of the propeptide in most glass sponge TGFβ ligands **(**Fig. 3f **)**, they all possess the characteristic RXXR cleavage site (except OmBMPLb) **(**Fig. 3c **)** that would allow the release of the predicted mature peptide and thus would activate TGFβ receptors. Most receptors display a typical domain composition, consisting in a signal peptide, a transmembrane domain (except OmActRII), a GS domain (in type I receptors only) and a kinase domain **(**Fig. 3f **)**, while the TGFβ-binding domain of the receptors is highlighted by a conserved cysteine pattern **(**Fig. 3f and Supplementary Figure 2 **)**. In addition, consistent with Smad proteins in other animals, the MAD homology 1 and the SMAD domain-like were found in all Smad proteins retrieved in glass sponges (Data not shown). Likewise, the DNA binding residues were predicted in Smad1/5 and Smad4 (but not in Smad2/3). In conclusion, all the elements (i.e. proteins and functional domains) necessary to transduce TGFβ signals into the nucleus are present in glass sponges, although some accessory proteins are missing **(**Fig. 3a **)**.

### Absence of key components of the Wnt pathway

Although conserved Notch and TGF-β pathways were retrieved in glass sponges, the situation was markedly different when considering the Wnt pathway since several key genes were entirely absent in both *A. vastus* and *O. minuta*. More specifically, we retrieved *apc*, *axin*, *β-catenin*, *frizzled B* (*fzdB*), *groucho, gsk3, LRP, porcupine* (porc; *only in O. minuta*) and *tcf/lef* but failed to identify *frizzled A* (*fzdA*), *dishevelled* (*dvl*), *wnt* and *wntless* (*wls*) **(**Supplementary Table 3 **)**.

In order to ensure that our observations faithfully reflect the glass sponge genome and not some technical issues, we searched for these 13 genes in various transcriptomes available for other sponge lineages: Homoscleromorpha (i.e *O. carmela* and *O. sp*), Calcarea (i.e. *L. complicata* and *S. ciliatum*) and Demospongiae (i.e. *E. muelleri*). We unambiguously identified these proteins in all transcriptomes **(**Table 2 and Supplementary Table 4–6 **)** and confirmed our findings by a KAAS analysis (Supplementary Figure 3)^29–31^. Consequently, we assume that the absence of Dvl, FzdA, Wls and Wnt in both glass sponge transcriptomes likely reflects their absence in their genomes.

**Table 2:** Presence / absence of the Wnt pathway components throughout metazoans.

	APC	Axin	B-cat	Dsh	Fzd	Gro	GSK3	LRP	Porc	TCF	Wls	Wnt	Refs
**Vertebrata**	•	•	•	•	•	•	•	•	•	•	•	•	^52^
**Urochordata**	•	•	•	•	•	•	•	•	•	•	•	•	^53^ *
**Platyhelminthes**	•	•	•	•	•	•	•	o	•	•	•	•	^54–57^
**Ecdysozoa**													
Arthropoda	•	•	•	•	•	•	•	•	•	•	•	•	^52^
Nematoda	•	•	•	•	•	•	•	o	•	•	•	•	^58^
**Cnidaria**													
Anthozoa	•	•	•	•	•	•	•	•	•	•	•	•	^22,59^
Myxozoa	o	o	•	o	o	•	•	o	o	•	o	o	^46^
**Placozoa**	o	•	•	•	•	•	•	•	•	•	•	•	^14^ *
**Ctenophora**	•	•	•	•	•	•	•	•	•	•	•	•	^15^
**Porifera**													
Hexactinellida	•	•	•	o	•	•	•	•	•	•	o	o	^22^ **
Demospongiae	•	•	•	•	•	•	•	•	•	•	•	•	^17,22^ **
Calcarea	•	o	•	•	•	•	•	•	•	•	•	•	^22,32^ **
Homoscleromorpha	•	•	•	•	•	•	•	•	•	•	•	•	^22,32^ **

Black dots correspond to proteins previously retrieved while a white dot means that the protein has not been evidenced until now. (*) means that some proteins were only retrieved on NCBI and/or Uniprot websites (i.e. no publication) while (**) imply that all components of the pathway were analyzed in the present study.

Beyond these four missing genes, five others encode proteins that lack key functional domains. Groucho, GSK3, LRP and Porc show a regular domain composition compared to their human orthologs **(**Fig. 4b,c **)**, whereas the others (APC, Axin, β-catenin, FzdB and TCF) do not. The region known to interact with β-catenin is not predicted in Axin proteins found in glass sponges **(**Fig. 4d **)**, and the APC protein is truncated, lacking the region allowing β-catenin, GSK3 and Axin binding **(**Fig. 4e **)**. This is consistent with previous analyses of Axin in demosponges (i.e. *Amphimedon queenslandica*), and APC proteins from demosponges and cnidarians (e.i. *Nematostella vectensis*)^17^. Thus, the formation of the β-catenin destruction complex (i.e. Axin, APC and GSK3) is questionable in non-bilaterian animals, especially when considering the absence of Axin protein in the genome of the calcarean *Sycon ciliatum* ^32^. Supporting this idea, Schippers and Nichols succeeded to identify adherent junction components (i.e. α-catenin and classical cadherin) but not Axin, APC or GSK3 as Emβcatenin binding partners by immunoprecipitation^33^. Nevertheless, chemical inhibitions suggest that Emβcatenin can be considered as a phosphorylation substrate of GSK3. Thus whether this phosphorylation involves the two other components and drives β-catenin degradation still requires further investigation in basal metazoans. The domain composition of FzdB, β-catenin and TCF is also unusual in glass sponges, even when compared to their demosponge orthologs: i) the Dvl-binding motif (KTXXXW) is absent/divergent in FzdB **(**Fig. 4f and g **)**, ii) the β-catenin shows a divergent GSK3-binding motif (Supplementary Figure 4), and lacks the Dvl-binding domains (i.e. PDZ-binding motif: S/TxV/L) **(**Fig. 4h **)**, iii) the β-catenin-binding domain of TCF is not predicted **(**Fig. 4h **)**.

The absence of three core proteins and the loss of some key functional domains or motifs in the remaining components support the idea that the Wnt pathway cascade cannot be considered as functional in syncytial glass sponges. We nevertheless envisage that the activation of Wnt pathway responsive genes may occur in glass sponges, but in a Wnt-independent way since the transcription factor Tcf/Lef and its co-repressor Groucho were characterized here.

### Analyses of the CEGs

We previously described the first transcriptome of *Oopsacas minuta* ^34^. Here we evaluated the completeness of several transcriptomic databases available not only for glass sponges (i.e. *Aphrocallistes vastus*) but also for other poriferan lineages (i.e. *Ephydatia muelleri*, *Leucosolenia complicata*, *Oscarella carmela*, *Oscarella sp*. and *Sycon ciliatum*) by searching the previously defined 248 core eukaryotic genes (CEGs)^35–38^. Despite high variability in the total number of base pairs among sponges (from 28.1 Mb to 68.6 Mb) **(**Table 3 **)**, a remarkably high number of CEGs (from 87.5% to 96.8%) was nonetheless recovered for most studied species (Supplementary Figure 5). Given the high percentage of CEGs present in each poriferan database, the collected datasets can be considered of high quality and that comparison between them is meaningful. Finally, only five CEGs absent in *O. minuta* (out of 14) are also missing in *A. vastus*, suggesting that taken together, these two transcriptomes likely reflect the overall genome of glass sponges.

**Table 3:** Comparative statistics of poriferan transcriptomes.

Species	Total	Contigs	N50	CEGs
*E. muelleri*	69.6 Mbp	85,971	1,324 bp	92.7%
*A. vastus*	64.7 Mbp	46,987	2,100 bp	96.8%
*O. minuta*	28.1 Mbp	34,421	914 bp	94.4%
*O. carmela*	58.5 Mbp	54,004	1,846 bp	94.8%
*O. sp*.	39.1 Mbp	172,354	472 bp	87.5%
*L. complicata*	80 Mbp	92,106	1,191 bp	93.1%
*S. ciliatum*	68.7 Mbp	50,731	1,959 bp	93.1%

Table providing the total size of each transcriptomes, the total number of contigs and the N50. The reliability of the two glass sponge transcriptomes (*A. vastus* and *O. minuta*) was compared to transcriptomes of sponges belonging to the other lineages (Demospongiae, Homoscleromorpha and Calcarea) by researching the 248 core eukaryotic genes (CEGs) previously defined as a proxy of genomes and transcriptomes completeness. Abbreviations: *A. vastus*, *Aphrocallistes vastus*; *E. muelleri*, *Ephydatia muelleri*; *L. complicata*, *Leucosolenia complicata*; *O. carmela*, *Oscarella carmela*; *O. sp*, *Oscarella sp*.; *O. minuta*, *Oopsacas minuta* and S*. ciliatum*, *Sycon ciliatum*.

## Discussion

Almost ten years ago, the acquisition of the first genome of sponges shed light on the conserved molecular toolkit of animal development, revealing that despite their apparent anatomical simplicity (i.e. few cell types and no organs), sponges share key developmental signaling pathways (i.e. Notch, TGFβ and Wnt modules) with more complex animals^6^. While the molecular characterization of these three modules has been investigated in *Amphimedon queenslandica* ^17,23,25^, comparative approaches in the other three poriferan lineages are still sparse^22,23,25,32^, especially for the deep sea phylum of glass sponges^22^. Briefly, our in depth analysis of two glass sponge transcriptomes supports that TGFβ and Notch signaling are likely functional in glass sponges. Indeed, we retrieved at least the minimal set of components required to transduce Notch and TGFβ signals from the membrane to the nucleus^39^, confirming the existence of both signaling in the last common ancestor of animals^6,23^. The present study also uses various transcriptomic databases to reveal the lack of the upper part of the Wnt signaling cascade in glass sponges.

In theory, it remains possible that our data reflect the absence of several Wnt pathway transcripts at the moment of sampling. This is, however, unlikely for multiple reasons: i) the analysis of CEGs allowed us to be highly confident regarding the coverage of the databases used in this study; ii) the exact same set of genes appeared to be absent in two independently acquired transcriptomes (i.e. Aix Marseille University, France and University of Alberta, Canada), while complete Notch and TGF-β modules were retrieved; iii) several paralogs of *wnt* and *dvl* genes (i.e. 3 to 21 *wnt* and 1 to 2 *dvl*) were previously described in other sponge lineages. Among them, some are dedicated to embryogenesis while some others are highly expressed in adult under homeostatic conditions^17,32,40^. Thus, by collecting adult specimens that contained numerous embryos at all developmental stages^41–43^, we should have easily retrieved at least some of these paralogous genes like it was the case for all other transcriptomes; iv) only a fraction of Wnt pathway gene transcripts were missing, while others were retrieved, suggesting that sampling did not skew our analysis.

Although the loss of a *bona fide* Wnt pathway in glass sponges clearly coincides with their atypical syncytial organization, it remains unclear whether both features are linked. Leys has argued that cytoplasmic streaming within the trabecular syncytium and through the pores of plugged junctions **(**Fig. 1e **)** may cause efficient but localized trafficking, allowing signals to be transmitted^19^. Thus, the syncytial organization of glass sponges could support signal transmission, making paracrine Wnt signaling through cell membranes dispensable. It is also interesting to note that the syncytial organization of glass sponges occurs following the fifth division (32-cell stage), when cells massively reorganize in a coordinated way^20^. In other animals, such as cnidarians and mammalians, the 32-cell stage marks the onset of Wnt pathway activation^44,45^. Therefore, the two outstanding features of glass sponges – the lack of a proper Wnt pathway and of a fully cellular organization – are most likely deeply interwined. We hypothesize that in this phylum, the acquisition of a syncytial organization with plugged junctions may have allowed an alternative mode of cell-to-cell communication during morphogenesis, thus bypassing the paracrine Wnt pathway and enabling its partial loss in the last common ancestor of glass sponges.

Our results, together with recent genomic and transcriptomic data on myxozoans^46^ – highly derived endoparasitic cnidarians devoid of Wnt pathway –, challenge the proposed universal importance of Wnt pathways in early development of animals^47^. Indeed, these unexpected findings suggest that the Wnt core (i.e. Wnt ligand secretion and Dsh translocation to the cell membrane) is needed neither to reach a multicellular stage, nor for cell differentiation and cell polarity (e.g. collar body of glass sponge; capsulogenic cells of myxozoans), nor for the acquisition of body axis polarity **(**Fig. 1h and i **)**. In contrast with the drastically simplified morphology of myxozoans with respect to the typical body plan of cnidarians, glass sponges have maintained a body plan comparable to other sponge lineages, making comparative approaches more relevant and easier to carry out. It is, however, striking to note that in both lineages the absence of the Wnt core coincides with the acquisition of a syncytial organization^19,48^, suggesting that the Wnt pathway is required only in truly multicellular organisms. These peculiar exceptions across the metazoan tree strengthen the idea that the Wnt pathway has played a key role in cell-to-cell communication during the acquisition of animal multicellularity.

In conclusion, despite the fact that animal multicellularity is related to the acquisition of three main developmental signaling pathways, only the TGFβ and Notch modules were conserved in glass sponges, whereas the transition to a syncytial organization may have caused the erosion of the Wnt pathway. In the future, it will be necessary to study the molecular basis of plugged junction establishment and whether these junctions allow transportation and exchange of signaling molecules.

## Methods

### Samples for microscopy

Specimens of *O. minuta* were fixed in a mixture of 2% osmium tetroxide, saturated with mercury chloride (6/1), desilicated in 5% hydrofluoric acid^43^. Thin sections were contrasted with 0.5% uranyl acetate and lead citrate and viewed in a Hitachi Hu 600 transmission electron microscopes. For scanning electron microscopy, specimens were fractured in liquid nitrogen after desilification, sputter-coated with gold palladium and observed under a Hitachi S-570 scanning electron microscope.

### Identification of orthologous genes

The Notch, TGF-β and Wnt pathway components were searched using a TBLASTN approach. To do so, sequences of *Mus musculus* were used as initial query and a 1.0 threshold e-value was applied in order to retrieve even highly divergent sequences. When no orthologous sequence was obtained, proteins already described in cnidarians and sponges were used as query. Orthologous assignations of candidate genes were done i) by reverse TBLASTN approach against NCBI database, ii) phylogenetic analyses and iii) domain analyses using Interproscan 5, epestfind as well as domain described in the literature^49,50^. In order to confirm our data concerning the Wnt pathway, we proceeded to an additional KEGG Automatic Annotation (http://www.genome.jp/tools/kaas/)^29–31^.

Sequence alignments for phylogenetic analyses were produced by ClustalW function of Bioedit. ADAM alignment was cut using Gblock software (parameters: 16/30; 16/30; 10; all) while TGFβ ligands, TGFβ receptors and Smad proteins analyses were restricted to the mature peptide (i.e. residues 287–378 of OmTGFLa); the protein kinase domain (i.e. residues 245–532 of OmTGFBRII) and the MAD/SMAD domains (i.e. residues 25–147 and 271–453 of OmSmad2/3) respectively. Bayesian analyses were performed with MrBayes (aamodel = wag; nchains = 4). 200,000 generations were sufficient for topological convergence in ADAM and Smad analyses while 3,000,000 and 5,000,000 generations were required for TGF-β ligands and TGF-β receptors, respectively.

### The 248 Core Eukaryotic Genes

A reciprocal best hits approach has been used to identify the 248 CEGs (Core Eukaryotic Genes)^35^ in sponge databases (Supplementary Table 7). To do so, the 248 cds of human CEGs^35,51^ were identified by TBLASTN (10E-100 threshold e-value) in the following database: Homo_sapiens.GRCh38.cds.all.fasta (ftp://ftp.ensembl.org/pub/release-80/fasta/homo_sapiens/cds/). The 248 human cds were used to identify candidates in sponges (TBLASTN, 10E-5 threshold e-value). Best hit were validated by a reciprocal approach against human (TBLASTN, 10E-5 threshold e-value). To do so, the 6 ORFs were generated for each poriferan candidate gene and the longest one was retained and used as query. Poriferan candidate genes were considered as orthologs to CEGs if the reverse best hit corresponded to the initial human cds or to a similar human sequence (coverage higher than 99% and e-value of 0.0). This allowed us to retrieve a large set of CEGs. Finally, phylogenetic analyses (alignment using ClustalW; ML analysis using PhyML 3.0, LG model and 1,000 bootstraps) and sequence identity matrixes (Bioedit software) allowed us to detect human in-paralogs (Supplementary Table 8) and thus to slightly increase the number of CEGs retrieved in each poriferan transcriptome.

### Data Availability Statement

All sequences described in the present study were deposited on NCBI website and the corresponding accession numbers are provided in the Supplementary Information file while full alignments and phylogenetic analyses are available upon request.

## Electronic supplementary material

Supplementary Information
