# Supplementary material for: Animal multicellularity and polarity without Wnt signaling
Source: Sci Rep. 2017 Nov 13;7:15383. doi: 10.1038/s41598-017-15557-5 (PMC5684314; doi:10.1038/s41598-017-15557-5)
Supplement: Supplementary file 1 — Supplementary Information [file 41598_2017_15557_MOESM1_ESM.doc]

SUPPLEMENTARY INFORMATION

**Animal multicellularity and polarity without Wnt signaling**

**Quentin Schenkelaars^a,b^,*, Marine Pratlong^a,c^, Laurent Kodjabachian^d^, Laura Fierro-Constain^a^, Jean Vacelet^a^, André Le Bivic^d^,*, Emmanuelle Renard^a,1^, Carole Borchiellini^a,1^,***

^a^ Aix Marseille Université, CNRS, IRD, IMBE UMR 7263, Avignon Université, Institut Méditerranéen de Biodiversité et d’Ecologie marine et continentale, Station Marine d’Endoume, Marseille, France

**^b^** Department of Genetics and Evolution, University of Geneva, Sciences III, 30 Quai Ernest Ansermet, CH-1211 Geneva 4, Switzerland

^c^ Aix Marseille Université, CNRS, Centrale Marseille, I2M, Equipe Evolution Biologique et Modélisation, Marseille, France

^d^ Aix Marseille Université, CNRS, Institute of Developmental Biology of Marseille (IBDM), case 907, 13288 Marseille cedex 09, France

^1^ These authors jointly supervised this work.

* Corresponding authors:

Schenkelaars Quentin: [quentin.schenkelaars@unige.ch](mailto:quentin.schenkelaars@unige.ch), +42 22 339 32 80

Carole Borchiellini [carole.borchiellini@imbe.fr](mailto:carole.borchiellini@imbe.fr), +33 49 104 16 11

André Le Bivic: [andre.le-bivic@univ-amu.fr](mailto:andre.le-bivic@univ-amu.fr), +33 49 126 97 41

Keywords: sponges, Wnt, Notch, TGF-beta, plug junction, syncytium

**RESULTS**

***A conserved Notch pathway***

**ST1:** Notch pathway in Hexactinellida…………………………………………………………….………p3

***A conserved TGF-β pathways***

**ST2**: TGF-β pathway in Hexactinellida…………………………………………….……..………….……p4

**SF1:** Deciphering TGFβ-like and BMP-like subfamilies using cysteine patterns……….p5

**SF2:** Conservation of TGFβ-binding in TGFβ receptor is revealed by conserved cysteines…………………………………………………………………….………………………………………….p6

***Absence of key components of the Wnt pathway***

**ST3:** Wnt pathway in Hexactinellida………………………………….……………………………….….p7

**ST4:** Wnt pathway in Calcarea…………………………………….…………………………………………p8

**ST5:** Wnt pathway in Demospongiae………………………………………….…….……………………p9

**ST6:** Wnt pathway in Homoscleromorpha…………………..…………………………………………p10

**SF3:** KEGG Automatic Annotation server (KAAS) analysis of the Wnt pathway in sponges………………………………………………………………………………………………………………….p11

**SF4:** Divergent GSK3-binding domains in β-catenin from glass sponges………..……….p12

***Quality control and reliability of the dataset***

**SF5:** Summary of the core eukaryotic genes in sponges…………………..……………..……..p12

**MATERIAL AND METHODS**

***Transcriptomic databases***

**ST7:** Publicly available poriferan databases……………………………………………………………p13

**ST9:** In-paralogous CEGs identified in Human………………………………………………………..p13

**RESULTS**

***A conserved Notch pathway***

**Supplementary Table 1: Notch pathway in Hexactinellida.** Proteins retrieved in the transcriptomes of *Aphrocallistes vastus* and *Oopsacas minuta.* Proteins that were not retrieved in glass sponges like in other sponge lineages are indicated in grey.

| Protein | TBLASTN best hit | Accession |
| --- | --- | --- |
| Av_FurinL | *Amphimedon queenslandica* Furin1L (0.0; XM_0114071778.2) | comp20467_c0_seq1 |
| Om_FurinL | *Amphimedon queenslandica* Furin1L (0.0; XM_0114071778.2) | MF589732 |
| Av_Notch | *Lingula anatina* EGF1 (2e-125; XM_013531508.1) | comp20880_c0_seq1 |
| Om_Notch | *Branchiostoma belcheri* Notch1L (6e-140; XM_019787970.1) | MF589733 |
| Av_Delta | *Sarcophilus harrisii* JAG2 (2e-111; XM_003756444.2) | comp20580_c0_seq1 |
| Om_Delta | *Sarcophilus harrisii* JAG2 (2e-111; XM_003756444.2) | KX932092 |
| Av_ADAM10 | *Apaloderma vittatum* ADAM10 (7e-125; XM_009872985.1) | comp17652_c0_seq1 |
| Om_ADAM10 | *Pundamilia nyererei* ADAM10L (3e-123; XM_005728239.2) | MF589734 |
| Av_ADAM17 | *A. queenslandica* ADAM10L (3e-125; XM019999824.1) | comp21139_c0_seq5 |
| Om_ADAM17 | *A. queenslandica* ADAM10L (1e-122; XM019999824.1) | MF589735 |
| Av_NCSTN | *Exaiptasia pallida* NCSTNL (7e-78; XM_021046965.1) | comp12430_c0_seq1 |
| Om_NCSTN | *Phascolarctos cinereus* NCSTN (3e-77; XM_020979556.1) | MF589736 |
| Av_PSEN2 | *Dipodomys ordii* PSEN2 (2e-97; XM_013017537.1) | comp16706_c0_seq1 |
| Om_PSEN2 | *Xenopus laevis* PSEN2L (1e-101; XM_018263074.1) | MF589737 |
| Av_PEN2 | *Mateseiulus occidentalis* PEN2 (3e-18; XM_003744478.2) | comp4385_c0_seq1 |
| Om_PEN2 | *Mateseiulus occidentalis* PEN2 (1e-14; XM_003744478.2) | MF589738 |
| Av_APH1 | *A. queenslandica* APH1bL (5e-17; XM_003382434.3) | comp7933_c0_seq1 |
| Om_APH1 | *A. queenslandica* APH1bL (2e-23; XM_003382434.3) | MF589739 |
| Av_MamL | Abs |  |
| Om_MamL | Abs |  |
| Av_SuH | *Caenorhabditis elegans* Lag-1 (1e-51; NM_001306810.1) | comp18879_c0_seq1 |
| Om_SuH | *Salmo salar* SuH (5e-41; XM_014155512.1) | MF589740 |
| Av_NCoRL | *Ciona intestinalis* NCoR2 (3e-34; XM_009861727.2) | comp21240_c0_seq7 |
| Om_NCoRL | *Ciona intestinalis* NCoR2 (4e-35; XM_009861727.2) | MF589741 |

***A conserved TGF-β pathways***

**Supplementary Table 2: TGF-β pathway in Hexactinellida.** Proteins retrieved in the transcriptomes of *Aphrocallistes vastus* and *Oopsacas minuta.* Proteins that were not retrieved in glass sponges like in other sponge lineages are indicated in grey.

| Protein | TBLASTN best hit | Accession |
| --- | --- | --- |
| Av_TGFBLa | Python bivittatus TGFB1 (2e-23; XM_007421491.1) | comp12871_c0_seq1 |
| Om_TGFBLa | Sinocyclochelius rhinocerous TGFB1 (3e-20; XM_016571614.1) | MF589721 |
| Av_TGFBLb | Clupea harengus GDF15 (2e-09; XM_012827770.1) | comp15811_c0_seq1 |
| Av_BMPLa | Oncopeltus fasciatus (4e-27; AY899334.1) | comp36903_c0_seq1 |
| Om_BMPLa | Euperipatoides rowelli Dpp (1e-30; KF195924.1) | MF589722 |
| Av_BMPLb | Agrilus planipennis Dpp (3e-27; XM_018469193.1) | comp15223_c1_seq1 |
| Om_BMPLb | Drosophila melanogaster Dpp (8e-32; XM_002021493.1) | MF589723 |
| Av_BMPLc | Notothenia coriiceps BMP6 (2e-06; XM_010780967.1) | comp8695_c0_seq1 |
| Om_BMPLc | Precis coenia Dpp (3e-06; L42141) | MF589724 |
| Av_ACVRII | Sparus aurata ACTVRIIB (2e-77; JF906099.1) | comp17823_c0_seq1 |
| Om_ACVRII | Scleropages formosus ACVR2b (3e-79; XM_018741689.1) | MF589725 |
| Av_TGFBRII | Cynoglossus semilaevis TGFBR2L (5e-49; XM_002612939.1) | comp19881_c0_seq1 |
| Om_TGFBRII | Branchiostoma floridae TGFBR2 (9e-50; HQ588928.1) | MF589726 |
| Av_ACVRI | Monopterus albus ACVR1L (8e-113;XM_020613991.1) | comp11929_c0_seq1 |
| Om_ACVRI | Echinops telfairi ACVRIIL1 (5e-60; XM_004711462.1) | MF589727 |
| Av_TGFBRIa | Galeopterus variegatus TGFBR1 | comp9449_c0_seq2 |
| Om_TGFBRIa | Orbicella faveolata TGFBR1 (8e-84; XM_020757298.1) | KX932092 |
| Av_TGFBRIb | Drosophila takahashii TGFBR1 (4e-95; EU249542.1) | comp17847_c0_seq1 |
| Om_TGFBRIb | Mustela sp. TGFBRI (2e-84; U37065.1) | MF589728 |
| Av_Smad1/5 | Opisthocomus hoazin Smad9 (0; XM009938116.1) | comp14593_c0_seq1 |
| Om_Smad1/5 | Orycteropus afer Smad5 (2e-151; XM_007943784.1) | MF589729 |
| Av_Smad2/3 | Branchiostoma bulcheri Smad2/3 (2e-136, HQ588924.1) | comp9980_c0_seq1 |
| Om_Smad2/3 | Priapulus caudatus Smad3L (1e-125: XM_014825211.1) | MF589730 |
| Av_Smad4 | Octodon degus Smad4 (5e-156; XM_004623663.1) | comp12820_c0_seq1 |
| Om_Smad4 | Octopus bimaculoides Smad4 (5e-161; XM_014924914.1) | MF589731 |
| Av_SARA | Abs |  |
| Om_SARA | Abs |  |
| Av_Ski/Sn | Abs |  |
| Om_Ski/Sn | Abs |  |
| Av_Smad6/7 | Abs |  |
| Om_Smad6/7 | Abs |  |

**HsLefty1**  **KLVR--FASQGAPAGLGEPQLELHTLDLGDYGAQGDCDPEAPMTEGTRCCR---QEMYIDLQ-GMKW**

**HsLefty2**  **KLVR--FASQGAPAGLGEPQLELHTLDLRDYGAQGDCDPEAPMTEGTRCCR---QEMYIDLQ-GMKW**

**HsTGFB1**  **RHRR--------------------------ALDTNYC----FSSTEKNCCV---RQLYIDFRKDLGW**

**HsTGFB2**  **RKKR--------------------------ALDAAYC----FRNVQDNCCL---RPLYIDFKRDLGW**

**HsTGFB3**  **RKKR--------------------------ALDTNYC----FRNLEENCCV---RPLYIDFRQDLGW**

**HsInhB**  **RRRR----------------------------RGLEC-----DGKVNICCK---KQFFVSFK-DIGW**

**HsMstn**  **RSRR---------------------------DFGLDC---DEHSTESRCCR---YPLTVDFE-AFGW**

**HsGDF11**  **RSRR---------------------------NLGLDC---DEHSSESRCCR---YPLTVDFE-AFGW**

**AvTGFBa**  **RSKR--------------------------AVTTVDC----SDPNVKLCCV---RPLTINFVDDLGW**

**OmTGFBa**  **RSRR--------------------------AVSSRYC----LGRNEKKCCV---RPLEINFKEDLGW**

**AvTGFBb**  **RDIR--------------------------QSLQNNC-----RSIDNNCCT---HPLNLNFA-DPLI**

**MlTGFBa**  **RMKR---------------------YGSRGGNSPQTC-----TNFPRQCCK---KDHWITFK-EVGL**

**MlTGFBb**  **RKKR----------------------------SGYDC---VNPRQQKKCCR---YHYFVKFA-DLGM**

**AqTGFB1L**  **RAKR--------------------QELPEGSTPLNLC-----KENQVTCCL---KPLNISFADDLGF**

**Aq_I1G3U8**  **RAKR----------------QSQVASATIPKLDTSFC---FPRPKEPNCCV---RTLTINFMRDLGV**

**HsBMP2**  **REKR------------------------------QAKHKQR-KRLKSSCKR---HPLYVDFS-DVGW**

**HsBMP4**  **RRRR--------------------------AKRSPKHHSQRARKKNKNCRR---HSLYVDFS-DVGW**

**HsGDF3**  **RKRR-----------------------AAIPVPKLSC--------KNLCHR---HQLFINFR-DLGW**

**HsGDF1**  **RPRR-------------------------------DAEPVLGGGPGGACRA---RRLYVSFR-EVGW**

**HsGDF5/BMP14** **RKRR--------------------------APLATRQGKRPSKNLKARCSR---KALHVNFK-DMGW**

**HsGDF6/BMP13** **RRRR--------------------------TAFASRHGKRHGKKSRLRCSK---KPLHVNFK-ELGW**

**HsGDF7**  **RRRRTALAGTRGAQGSGGGGGGGGGGGGGGGGGGGGAGRGHGRRGRSRCSR---KSLHVDFK-ELGW**

**HsBMP5**  **RSVR--------AANKRKNQNRNKSSSHQDSSRMSSVGDYNTSEQKQACKK---HELYVSFR-DLGW**

**HsBMP6**  **RTTR-------SASSRRRQQSRNRSTQSQDVARVSSASDYNSSELKTACRK---HELYVSFQ-DLGW**

**HsBMP7**  **RSIR-------STGSKQRSQNRSKTPKNQEALRMANVAENSSSDQRQACKK---HELYVSFR-DLGW**

**HsBMP8B**  **RTPR-------AVRPLRRRQPKKSNELPQANRLPGIFDDVHGSHGRQVCRR---HELYVSFQ-DLGW**

**HsNodal**  **RHRR-----------------------------------HHLPDRSQLCRK---VKFQVDFN-LIGW**

**HsGDF10/BMP3** **RKDR--------KKKDQDTFTAASSQVLDFDEKTMQKARRRQWDEPRVCSR---RYLKVDFA-DIGW**

**HsGDF2/BMP9**  **RRKR-------------------------------------SAGAGSHCQK---TSLRVNFE-DIGW**

**HsGDF9**  **RHRR-----------GQETVSSELKKPLGPASFNLSEYFRQFLLPQNECEL---HDFRLSFS-QLKW**

**AvBMPLa**  **RVRR-----------------------------SSPSTSRDTSHTSSQCQN---HHLYISFR-EIGW**

**OmBMPLa**  **RNRR------------------------------SSNDIPDYPPDSDQCQK---HNLYISFN-EIGW**

**OmBMPLb**  **SSNR-----------------------NSDPTETPPWLQGTSSSASTSCQR---YSNQINFD-AIGW**

**AvBMPLb**  **RSRR-------------------------PWEPDEDNPSSENPAPVTNCQR---YSHVIDIEEDLGW**

**OmBMPLc**  **REVR----------------------------------------ASSSCSRVSQSMKVEEFLKIMRT**

**AvBMPLc**  **RNVR----------------------------------QVMQQNIYSPCSRVSKNIKVRQFLKIFQS**

**MlBMP5-8**  **RDTR----------------------------NSQSLDSINYDPSQSHCQR---KELFIDFS-RLGW**

**MlBMP3**  **KSAR-----------------------RHFLQMFGETSHIERRSASPHCRV---RNLMVNFN-KIGL**

**MlTGF1a**  **RTKR---------------------RAKNWATPLLYRDVTELEKVNYHCRR---QDLWINFD-EIGW**

**MlTGF1b**  **RKKR-----------------GTAGFSPTSHHTIQYKDVTPLEVPAFPRQK---VKFDLNLV-DIGW**

**MlTGF2**  **RAAVR----------------------------DEDEGVSASRGFQHRCTM---MEFYVNFT-KLGW**

**MlTGF3**  **RQSK-----RKVVNNFTHRYDNPRTPATKFNEPSYWMNPPDQDQDEGKCGV---RLLYLQFE-KDMS**

**MlTGF4**  **RSHR----------------ARDRFRETAIIHSPFERSTKSSKFGGEKCKL---EPLYINFR-DLGW**

**AqBMP2L**  **RRKR---------------------------------ETYSKPKDFGICKL---HTYHVNFT-SLGW**

**AqBMP6L**  **RGKR-------------------------------------SSNDENACKI---HNLTVYFQ-QIQL**

**Aq_I1F3M5**  **RKSR-----------------------------------------SKHCRL---VRHYIDFK-RIGF**

**Aq_I1G5C7**  **KSKR-------------------------SAQPTESTSKIHPVNKTIPCHL----IPYQ----VIG-**

**Aq_I1FJ72**  **REAR----------------------------GISTNKNDTAEEKPSECPV---HDLEVSLS-SI-L**

**AqTGFB**  **RTRR-------------------------------STGKTVSKGDSEECSK---SSLSIDKG-QLAQ**

**AqBMP4**  **RNTR------------------------------------SSGTSNGECRK---SELTITKE-QLGQ**

**Supplementary Figure 1: Deciphering TGFβ-like and BMP-like subfamilies using cysteine patterns.** Alignment of the residues allowing the discrimination between TGFβ-like and BMP-like subfamilies. In blue, the glass sponge sequences; in red *Mnemiopsis leidyi* (Ml) proteins and in green the sequences described in *Amphimedon queenslandica* (Demospongiae).

**Hsa_Actr1**  **LYMCVCEGLS-------CGNE----------------------DHCEGQ--------------QCFSSLS-------INDGFHVYQKGCF**

**Nv 178197**  **VYRCICSD---------CRSNAGE-------------------QTCSTE-------------TGCFSSLF------LLENGQSSVTKGCL**

**Hsa_BMPr1B**  **VLRCKCHHH--------CPEDSVN-------------------NICSTD-------------GYCFTMIE------EDDSGLPVVTSGCL**

**Nv 165860**  **AIRCKCSEHS-------CPGDRIN-------------------DTCTTT-------------GKCYKKIA-------EEEGYELITYGCL**

**Hsa_TGFbr1** **ALQCFCHL---------CTKDNFT---------------------CVTD-------------GLCFVSV-------TETTDKVIHNSMCI**

**Nv_140805**  **QITCKCDY---------CQETNST---------------------CVTK-------------GACLTILQR-----DSSTGEVKRGHACT**

**AvTGFBRIA** **SLKCYCANDGQR----FCDLAYDA---------------------CNTS-------------GICVGELV-------KHELINIIVQSCL**

**OmTGFBRIA** **PLICHCSPDS-------C-YDYPT---------------------CITE-------------GLCIEQVI-------GSELT-STTQGCV**

**AvTGFBRIB** **QLECHAYVSGEP---NVCTVSYDP-------------------KLDQTP-------------PKCYVVEDFETEEAFYARKPDRFEQGCV**

**OmTGFBRIB** **ELTCIRKISNTQ---DTCTVTYNK-------------------DIHPTP-------------PRCLVVEDYETDSAFNARIPDRFEQGCV**

**AvACVRI**  **NITCVCNYTNSEV---TCNPDN----------------------TCIG--------------LICFKTVK-------IINSVIYNEDGCI**

**OmACVRI**  **EINCTCKHTAQDI--GECSSND----------------------TCIG--------------FVCYVFTR-------RYLHSISTQWGCL**

**Hsa_Actr2**  **TRECIYYNANWEL---ERTNQ-------------------SGLERCEGEQDK---------RLHCYASWA------NSSGTIELVKKGCW**

**Nv act2**  **LQTCESFGQL-------CHETGE----------------CNNTIVCNFRES------------HCYTLWE------NTTEGVNIIKKGCF**

**AvACVRII**  **SNTCYFKNINET-----CNEESAS---------------CLYIKSCEEFFEENDSGQLSDQHTHCFILYD------TINGATVPINRGCF**

**OmACVRII**  **TNLCFFKDSNATSG---CELNNS----------------CLHIDSCQVGLPDNQQEQ--SEHTHCFLYYLLN----GPNXLTLPLARGCF**

**Hsa_BMPr2**  **ERLCAFKDPYQQDL--GIGESRISH--------------ENGTILCSK-------------GSTCYGLWE------KSKGDINLVKQGCW**

**Hsa_TGFbr2** **------------------------------------------~MIL-------------PLHNLGNGVRSHNFSF-LYFILEDAASPKCI**

**AvTGFBRII** **SILCLSCRNNRNFT---CRQDYISEDRGFSRYEC--TFVCDTIVNC-------------TSIPYCNAFMN-----KVSTQNSYWSSQFCQ**

**OmTGFBRII** **AVICYTCANNRDIT---CRLLELTADNGFSRYEC--TANCNQTEDC----------------PYCRVLLT----NTKSENFYKMTYIGCP**

**Hsa_Actr1**  **QVYEQG-------KMTCKTPPSPGQ----------------------------------AVECCQG-DWC**

**Nv 178197**  **KDADHY-------NMMCRGPRGKPN------------------------------------RCCTH-DLC**

**Hsa_BMPr1B** **GLEGS--------DFQCRDTPIPHQ--------------------------------RRSIECCTERNEC**

**Nv 165860**  **PPEEQT-------DMQCNTPASVHR-------------------------------NKISVLCCNNRDLC**

**Hsa_TGFbr1** **AEIDLI---PRDRPFVCAPSSKTGS--------------------------------VTTTYCCNQ-DHC**

**Nv_140805**  **YN-------PPADRLVCEPGYSTER-------------------------------PHQLYHCCYY-NMC**

**AvTGFBRIA** **PEEEIA--------NSCNELNDIFR----------------------------------TRLCCHE-NFC**

**OmTGFBRIA** **PPDLIA--------STCSVYSTIHR------------------------------------FCCDT-YLC**

**AvTGFBRIB** **GGSTLS-----SSAFFCNGKSQQIFDY-------------------------QSMKYVEYTYCCDY-DYC**

**OmTGFBRIB** **GGDTGD-----SSEFHCDTPNQRIRIY-------------------------NQRKYVEYTYCCST-DLC**

**AvACVRI**  **SHHNS-DPDTQSPDFQCSQSQDYI------------------------------KSPKHQTDCCCT-DGC**

**OmACVRI**  **KEDDIAEASHISNDSLCPRKFPFE------------------------------YSPNLLDECCCT-DYC**

**Hsa_Actr2**  **LDDF-----NCYDRQECVA---TE-------------------------------ENPQVYFCCCEGNFC**

**Nv act2**  **IRNHLE----CRGDHQCLGTPRTK-------------------------------GGRTVFFCCCSDHFC**

**AvACVRII**  **SAPG------CE-ENNCHTASHSK------------------------------GL---QLYCCCMGDLC**

**OmACVRII**  **DHKG------CD-DNVCYRRAPSE------------------------------SIPYPQLYCCCKGNLC**

**Hsa_BMPr2**  **SHIGDPQ--ECH-YEECVVTTTPPS-----------------------------IQNGTYRFCCCSTDLC**

**Hsa_TGFbr2** **MKEK-------------KKPGET------------------------------------FFMCSCSSDEC**

**AvTGFBRII**  **RT-M------FSPNATCATSTSRYFANSSYIQQLFTRDYVNTSLLPG------PISNFPNVFCGCNTSYC**

**OmTGFBRII** **SPGR------LDLNDTCSATNSVEFSNSSYILSLFTQNYMNTSLLPQNTSDPRRSTIFPPFLCACNTSYC**

**Supplementary Figure 2: Conservation of TGFβ-binding in TGFβ receptor is revealed by conserved cysteines.** The cysteine residues conserved in almost all proteins are indicated in black while the others are indicated in red. In blue, the glass sponge sequences.

***Absence of key components of the Wnt pathway***

**Supplementary Table 3: Wnt pathway in Hexactinellida. Proteins retrieved in the transcriptomes of *Aphrocallistes vastus* and *Oopsacas minuta.* Proteins that were not retrieved in glass sponges but found in other poriferan lineages are indicated in red. *No Wnt proteins were retrieved in *A. vastus* and *O. minuta* while 31, 29, 3, 11 and 10 contigs coding for Wnt proteins were found in *L. complicate, S. ciliatum*, *E. muelleri, O. carmela* and *O. sp* transcriptomes, respectively.**

| Protein | TBLASTN best hit | Accession |
| --- | --- | --- |
| Av_APC | Acropora digitifera APC2L (5e-42; XM_015911078.1) | comp17952_c0_seq1 |
| Om_APC | Nematostella vectensis APC (3e-46; KT381584.1) | KU316198 |
| Av_Axin | Picoides pubescens RGS18 (3e-08; NM_009909105.1) | comp13763_c0_seq1 |
| Om_Axin | Latimeria chalumnae RGS18 (5e-07; XM_006007512.1) | KX905244 |
| Av_Bcat | Crepidula fornicata βcat (4e-128; HM040900.1) | comp17679_c0_seq1 |
| Om_Bcat | Chelonia mydas Jup (2e-113; XM_007054712.1) | KU316199 |
| Av_Dsh | Abs |  |
| Om_Dsh | Abs |  |
| Av_FzdA | Abs |  |
| Om_FzdA | Abs |  |
| Av_FzdB | Oopsacas minuta FzdB (5e-75; KM365035.1) | comp96115_c0_seq1 |
| Om_FzdB | Suberites domuncula Fzd (1e-25; AJ566637.1) | AJE25510.1 |
| Av_Gro | Pygoscelis adeliae TLE1 (1e-117; XM_009331843.1) | comp25886_c0_seq1 |
| Om_Gro | Pediculus humanus corporis Gro (3e-127; XM_002427207.1) | KU316200 |
| Av_GSK3 | Rana dybowskii GSK3 (0; KU986657.1) | comp17686_c0_seq3 |
| Om_GSK3 | Rana dybowskii GSK3 (0; KU986657.1) | KU316201 |
| Av_LRP | Branchiostoma belcheri Lrp6 (3e-71; XM_019784403.1) | comp18119_c0_seq1 |
| Om_LRP | Gekko japonicus Lrp6 (5e-74; XM_015409520.1) | KU316202 |
| Av_Porc | Abs |  |
| Om_Porc | Aethina tumida Porc (3e-14; XM_020017250.1) | KU316203 |
| Av_TCF | Nematostella vectensis Tcf-Lef (7e-50; DQ497247.1) | comp24352_c0_seq1 |
| Om_TCF | Amphimedon queenslandica Tcf (8e-44; NM.001305248.1) | KU316204 |
| Av_Wls | Abs |  |
| Om_Wls | Abs |  |
| Av_Wnt* | Abs |  |
| Om_Wnt* | Abs |  |

***Supplementary Table 4: Wnt pathway in Calcarea.*** *Proteins retrieved in the transcriptomes of Leucosolenia complicata and Sycon ciliatum. Proteins that were not retrieved in glass sponges are indicated in blue. *No axin protein was retrieved in the genome of Sycon ciliatum^1^ confirming the results obtained from transcriptomic databases. **31 and 29 contigs matching with Wnt proteins were retrieved here in L. complicata and S. ciliatum transcriptomes, respectively. However, we provide here only one of them*.

| Protein | TBLASTN best hit | Accession |
| --- | --- | --- |
| Lc_APC | Drosophila miranda APCL (1e-24; XM_017287481.1) | lctid33567| |
| Sc_APC | Drosophila miranda APCL (1e-27; XM_017287481.1) | sctid30022| |
| Lc_Axin* | Abs |  |
| Sc_Axin* | Abs |  |
| Lc_Bcat | Sycon ciliatum βcatA (0; HG973379.1) | lctid45409| |
| Sc_Bcat | Sycon ciliatum βcatA (0; HG973379.1) | sctid33494| |
| Lc_Dsh | Sycon ciliatum DvlA (0; HG973375.1) | lctid14181| |
| Sc_Dsh | Sycon ciliatum DvlA (0; HG973375.1) | sctid23133| |
| Lc_FzdA | Sycon ciliatum FzdA (2e-28; HG973370.1) | lctid52861| |
| Sc_FzdA | Sycon ciliatum FzdA (0; HG973370.1) | sctid36859| |
| Lc_FzdB | Sycon ciliatum FzdB (0; HG973371.1) | lctid20061| |
| Sc_FzdB | Sycon ciliatum FzdB (0; HG973371.1) | sctid54944| |
| Lc_Gro | Homo sapiens TLE4 (2e-93; M99439.1) | lctid55252| |
| Sc_Gro | Homo sapiens TLE4 (3e-108; M99439.1) | sctid37416| |
| Lc_GSK3 | Lepisosteus oculatus GSK3 (0; XM_006639046.2) | lctid72823| |
| Sc_GSK3 | Ictalurus punctatus GSK3L (0; XM_017457416.1) | sctid71625| |
| Lc_LRP | Sycon ciliatum LRP5/6 (0; HG973374.1) | lctid7793| |
| Sc_LRP | Sycon ciliatum LRP5/6 (0; HG973374.1) | sctid6877| |
| Lc_Porc | Strongylocentrotus purpuratus Porc (3e-91; XM_011663897.1) | lctid53766| |
| Sc_Porc | Strongylocentrotus purpuratus Porc (2e-97; XM_011663897.1) | sctid49708| |
| Lc_TCF | Sycon ciliatum TCFA (1e-72; HG973377.1) | lctid43964| |
| Sc_TCF | Sycon ciliatum TCFA (0; HG973377.1) | sctid25941| |
| Lc_Wls | Acropora digitifera Wls (3e-73; XM_015901733.1) | lctid77986| |
| Sc_Wls | Acropora digitifera Wls (2e-74; XM_015901733.1) | sctid24281| |
| Lc_Wnt** | Sycon ciliatum WntE (2e-175; HG973353.1) | lctid93240| |
| Sc_Wnt** | Sycon ciliatum WntT (0; HG973368.1) | sctid77575| |

**Supplementary Table 5: Wnt pathway in Demospongiae. Proteins retrieved in the transcriptomes of *Ephydatia muelleri.* Proteins that were not retrieved in glass sponges are indicated in blue.*3 contigs matching with Wnt proteins were retrieved here in *E. muelleri* transcriptome*.* However, we provide here only one of them*.***

| Protein | TBLASTN best hit | Accession |
| --- | --- | --- |
| Em_APC | Amphimedon queenslandica APC (7e-144; NM_001279303.1) | comp57850_c0_seq3 |
| Em_Axin | Amphimedon queenslandica Axin (2e-56; NM_001279315.1) | comp42350_c0_seq1 |
| Em_Bcat | Amphimedon queenslandica βcat (0; NM_001279305.1) | comp60477_c0_seq1 |
| Em_Dsh | Ephydatia muelleri Dsh (0; KM983305.1) | comp70341_c0_seq1 |
| Em_FzdA | Ephydatia muelleri FzdA (0; KM365038.1) | comp70121_c0_seq1 |
| Em_FzdB | Ephydatia muelleri FzdB (0; KM365039.1) | comp66535_c0_seq1 |
| Em_Gro | Amphimedon queenslandica Gro (0; NM_001279307.1) | comp62186_c0_seq1 |
| Em_GSK3 | Amphimedon queenslandica GSK3 (0; NM_001279283.1) | comp62965_c0_seq1 |
| Em_LRP | Vicugna pacos LRP6 (3e-90; XM_015235650.1) | comp61891_c0_seq1 |
| Em_Porc | Amphimedon queenslandica Porc (2e-119; XM_003387023.2) | comp61100_c0_seq4 |
| Em_TCF | Suberites domuncula TCF (2e-121; AJ304864.1) | comp38874_c0_seq1 |
| Em_Wls | Thamnophis sirtalis Wls (3e-78; XM_014066442.1) | comp64939_c0_seq1 |
| Em_Wnt* | Ephydatia muelleri Wnt (0; HM363029.1) | comp54380_c0_seq8 |

**Supplementary Table 6: Wnt pathway in Homoscleromorpha. Proteins retrieved in the transcriptomes of *Oscarella carmela* and *Oscarella sp.* Proteins that were not retrieved in glass sponges are indicated in blue.*11 and 10 contigs matching with Wnt proteins were retrieved in *O. carmela* and *O. sp.* transcriptomes*,* respectively. However, we provide here only one of them*.***

| Protein | TBLASTN best hit | Accession |
| --- | --- | --- |
| Oc_APC | Octopus bimaculoides APC (8e-88; XM_014934454.1) | comp56915_c0_seq1 |
| Osp_APC | Trichogramma pretiosum APC (7e-46; XM_014373972.1) | comp37901_c0_seq1 |
| Oc_Axin | Copidosoma floridanum Loco (3e-14; XM_014352820.1) | comp23540_c0_seq2 |
| Osp_Axin | Solenopsis invicta Loco (6e-17; XM_011162315.1) | comp18915_c0_seq1 |
| Oc_Bcat | Oscarella carmela βcat (0; HQ234356.1) | comp41806_c0_seq1 |
| Osp_Bcat | Oscarella sp βcat (0; HQ234359.1) | comp3163_c0_seq1 |
| Oc_Dsh | Oscarella lobularis Dsh (0; KT898949.1) | comp44599_c0_seq1 |
| Osp_Dsh | Oscarella lobularis Dsh (0; KT898949.1) | comp18085_c0_seq1 |
| Oc_FzdA | Oscarella carmela FzdA1 (0; KM365032.1) | comp18259_c0_seq1 |
| Osp_FzdA | Oscarella carmela FzdA1 (0; KM365032.1) | comp4918_c0_seq1 |
| Oc_FzdB | Oscarella carmela FzdB (0; KM365034.1) | comp51684_c0_seq1 |
| Osp_FzdB | Oscarella lobularis FzdB (5e-156; KM365031.1) | comp13598_c0_seq1 |
| Oc_Gro | Saccoglossus kowalevskii Gro (0; NM_001165025.1) | comp37815_c0_seq2 |
| Osp_Gro | Saccoglossus kowalevskii Gro (0; XM_006817688.1) | comp5560_c0_seq1 |
| Oc_GSK3 | Lepidothrix coronata GSK3 (0; XM_017814730.1) | comp23762_c0_seq3 |
| Osp_GSK3 | Serinus canaria GSK3 (0; XM_009102590.1) | comp1777_c0_seq1 |
| Oc_LRP | Odobenus rosmarus LRP4 (0; XM_004399546.1) | comp36158_c0_seq6 |
| Osp_LRP | Ficedula albicollis LRP4 (0; XM_016298907.1) | comp9961_c0_seq1 |
| Oc_Porc | Strongylocentrotus purpuratus Porc (8e-101; XM_011663897.1) | comp20806_c0_seq1 |
| Osp_Porc | Hydra vulgaris Porc (6e-29; XM_012708030.1) | comp135033_c0_seq1 |
| Oc_TCF | Nematostella vectensis TCF-LEF (1e45; DQ497247.1) | comp28653_c0_seq3 |
| Osp_TCF | Nematostella vectensis TCF-LEF (3e41; DQ497247.1) | comp21846_c0_seq1 |
| Oc_Wls | Saccoglossus kowalevskii Wls (3e-86; XM_001634582.1) | comp35981_c0_seq4 |
| Osp_Wls | Nematostella vectensis Wls (2e-37; AB274034.1) | comp8841_c0_seq1 |
| Oc_Wnt | Oscarella lobularis Wnt1 (2e-120; GQ144646.1) | comp8600_c0_seq1 |
| Osp_Wnt | Chelonia mydas Wnt4 (3e-51; XM_007061391.1) | comp8090_c0_seq1 |

**
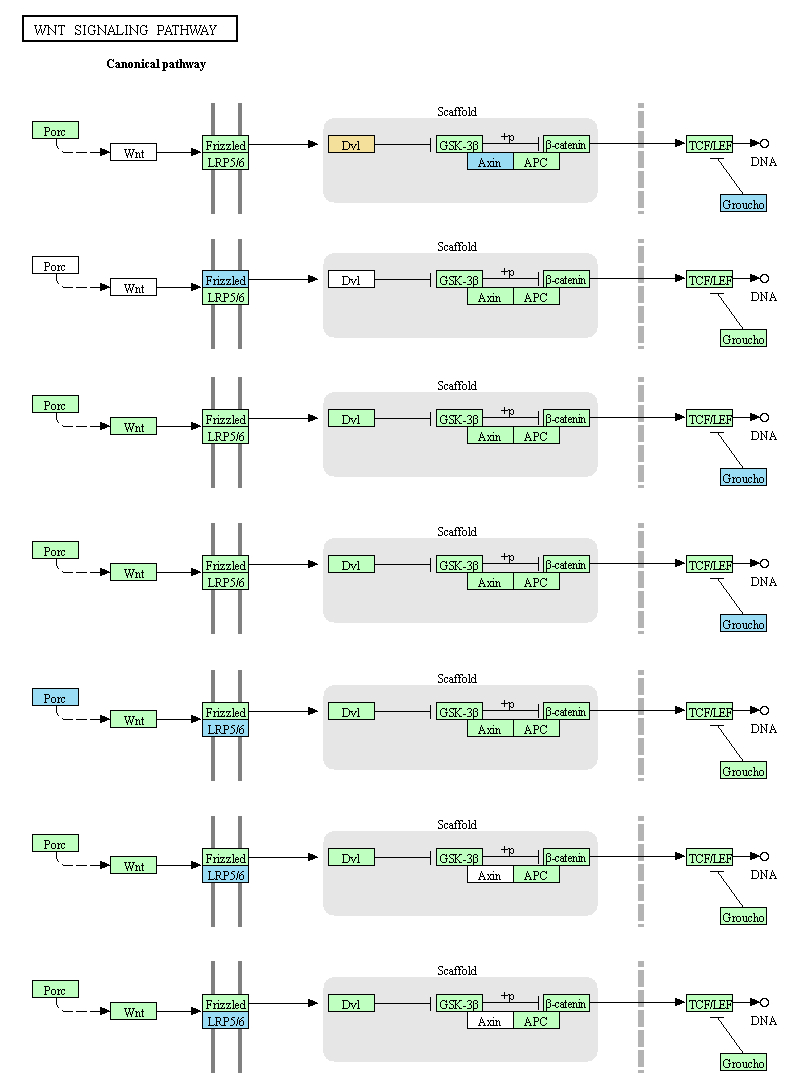
**

*Oopsacas minuta*

*Aphrocallistes vastus*

*Ephydatia muelleri*

*Oscarella carmela*

*Oscarella sp.*

*Sycon ciliatum*

*Leucosolenia complicata*

**Supplementary Figure 3: KEGG Automatic Annotation server (KAAS) analysis of the Wnt pathway in sponges.** KAAS ^29-31^ allowed us to confirm the absence of Wnt and Dishevelled (Dvl) in glass sponges (white) while they were retrieved in all other poriferan transcriptomic databases (green). Note that the Dvl protein found in *O. minuta* (s_pon_c10095) using the KAAS server actually belongs to the Dixin family (orange). Several proteins were identified only manually (blue), confirming the necessity of the manual approach.

*
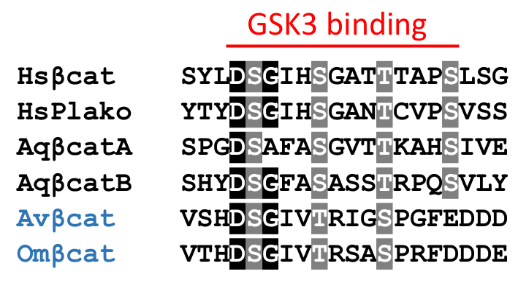
*

**Supplementary Figure 4: Divergent GSK3-binding domains in β-catenin from glass sponges.** The S/TxxxS/TxxxS/TxxxS/T motif described as Dsh-binding domain is retrieved in *Amphimedon queenslandica* (grey) but the final residue is absent in both glass sponges.

***Quality control and reliability of the dataset***


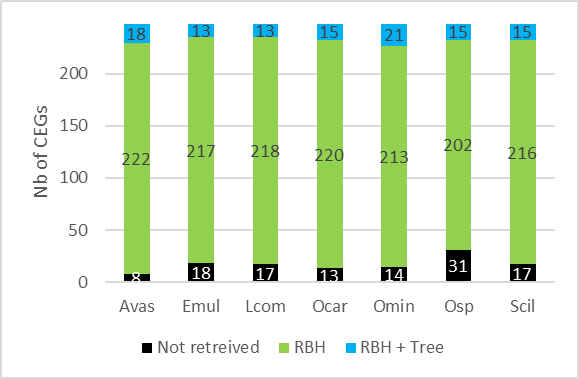


**Supplementary Figure 5: Summary of the core eukaryotic genes in sponges**. Number of CEGs retrieved using Reciprocal Best Hit approach (RBH) or RBH plus phylogenetic analyses (RBH + Tree) in each poriferan transcriptome. Abbreviations: Avas, *Aphrocallistes vastus*; Emul, *Ephydatia muelleri*; Lcom, *Leucosolenia complicata*; Ocar, *Oscarella carmela* ; Osp, *Oscarella sp*. and Scil, *Sycon ciliatum*. Further information concerning the CEGs retrieved in each species are available upon request.

**MATERIAL AND METHODS**

***Transcriptomic databases***

**Supplementary Table 7: Publicly available poriferan databases.**

| Lineage | Species | Website |
| --- | --- | --- |
| Calcarea | *Leucosolenia complicata* | http://www.compagen.org/ |
|  | *Sycon ciliatum* | http://www.compagen.org/ |
| Demospongiae | *Ephydatia muelleri* | http://www.compagen.org/ |
| Hexactinellida | *Aphrocallistes vastus* | https://era.library.ualberta.ca/ |
|  | *Oopsacas minuta* | Personal database |
| Homoscleromorpha | *Oscarella carmela* | http://www.compagen.org/ |
|  | *Oscarella sp.* | http://www.compagen.org/ |

**Supplementary Table 8: In-paralogous CEGs identified in Human.** Human in-paralogous CEGs were revealed by phylogenetic analyses (available upon request).

| KOG | Best hit (HsCds0) | In-paralogs (HsCds1) |  | KOG | Best hit (HsCds0) | In-paralogs (HsCds1) |
| --- | --- | --- | --- | --- | --- | --- |
| KOG0211 | ENST00000527614 | ENST00000462990 |  | KOG1643 | ENST00000396705 | ENST00000613953 |
|  |  | ENST00000322088 |  | KOG0419 | ENST00000371558 | ENST00000265339 |
| KOG3405 | ENST00000442738 | ENST00000606538 |  | KOG1769 | ENST00000392245 | ENST00000332859 |
|  |  | ENST00000470701 |  |  |  | ENST00000420826 |
| KOG1532 | ENST00000610189 | ENST00000458167 |  | KOG3189 | ENST00000268261 | ENST00000216259 |
| KOG2606 | ENST00000338352 | ENST00000404789 |  | KOG3205 | ENST00000580685 | ENST00000536592 |
| KOG1688 | ENST00000378512 | ENST00000443438 |  |  |  | ENST00000541546 |
|  |  | ENST00000488353 |  | KOG2792 | ENST00000535425 | ENST00000255390 |
| KOG1367 | ENST00000304801 | ENST00000373316 |  | KOG0225 | ENST00000295266 | ENST00000422285 |
| KOG0985 | ENST00000617103 | ENST00000393043 |  |  |  | ENST00000379806 |
|  |  | ENST00000269122 |  | KOG1393 | ENST00000369406 | ENST00000433297 |
|  |  | ENST00000621829 |  | KOG1770 | ENST00000469257 | ENST00000232905 |
| KOG4392 | ENST00000613744 | ENST00000292614 |  | KOG1439 | ENST00000380191 | ENST00000447750 |
| KOG1637 | ENST00000335968 | ENST00000265112 |  | KOG1373 | ENST00000243253 | ENST00000304267 |
| KOG0359 | ENST00000275603 | ENST00000436961 |  |  |  | ENST00000298428 |
| KOG1458 | ENST00000375337 | ENST00000415431 |  | KOG1088 | ENST00000544844 | ENST00000308774 |
| KOG0888 | ENST00000393190 | ENST00000393196 |  | KOG0077 | ENST00000439578 | ENST00000431664 |
| KOG0175 | ENST00000361611 | ENST00000438442 |  | KOG1533 | ENST00000374135 | ENST00000228827 |
| KOG3499 | ENST00000533498 | ENST00000584577 |  | KOG0937 | ENST00000250244 | ENST00000291439 |
